# *BAG*家族基因表达与肺腺癌易感性的相关性研究

**DOI:** 10.3779/j.issn.1009-3419.2010.10.03

**Published:** 2010-10-20

**Authors:** 颖 李, 红雨 刘, 竞 王, 永文 李, 蘅 吴, 欣欣 杜, 伟强 王, 蛟 岳, 清华 周, 军 陈

**Affiliations:** 300052 天津，天津医科大学总医院，天津市肺癌研究所，天津市肺癌转移与肿瘤微环境重点实验室 Tianjin Key Laboratory of Lung Cancer Metastasis and Tumor Microenvironment, Tianjin Lung Cancer Institute, Tianjin Medical University General Hospital, Tianjin 300052, China

**Keywords:** 肺肿瘤, *BAG*家族基因, 危险因素, Lung neoplasms, Bcl2-associated athanogene, Risk factors

## Abstract

**背景与目的:**

BAG（Bcl-2-associated athanogene）家族是一个多功能的抗凋亡家族，其表达与多种肿瘤的发病及预后相关。*BAG*基因是近年发现的抗凋亡基因家族，调节各种生理过程，包括细胞凋亡、肿瘤发生、神经分化、应激反应和细胞周期等。BAG家族的表达状态与某些肿瘤的发病和预后相关。本研究的目的是探讨*BAG*家族基因表达水平与肺腺癌易感性的关系，在分子水平探讨肺腺癌发病的相关危险因素。

**方法:**

采用基因表达谱芯片技术检测29例肺腺癌患者癌组织及癌旁肺组织*BAG*家族基因的表达，应用配对*t*检验和*Cox*回归拟合单因素条件*Logistic*回归方法。并利用美国GEO数据库验证相关结果。

**结果:**

*BAG-1*、*BAG-2*、*BAG-5*基因癌旁肺组织中表达高于癌组织且为肺腺癌发生的保护因素（*P* < 0.05, OR < 1）；*BAG-4*基因在癌组织中表达高于癌旁肺组织且为肺腺癌发生的危险因素（*P* < 0.05, OR>1）；*BAG-3*基因在癌组织及癌旁正常组织表达差别无统计学意义（*P*>0.05）。

**结论:**

*BAG-1*、*BAG-2*、*BAG-5*基因可能是为肺腺癌发生的保护性因素，*BAG-4*基因可能是为肺腺癌发生的危险性因素。

## Abstract (en)

**Background and objective:**

*BAG* genes (Bcl-2-associated athanogene) belong to a recently discovered multifunctional anti-apoptosis gene family that regulate various physiological processes which include apoptosis, tumorigenesis, neural differentiation, stress response and cell cycle and so on. The expression status of *BAG* family genes are related to certain tumor incidence and prognosis. The aim of this study is to explore the association of the *BAG* family gene expression status with the susceptibility of lung adenocarcinoma.

**Methods:**

The gene expression data of *BAG* family genes from 29 cases of lung adenocarcinoma tissues and matched pericancerous lung tissess were generated by microarray chips. *Cox* regression was used to analyze the association between the expression of *BAG* family genes and the susceptibility of lung adenocarcinoma and the results were verified by GEO database.

**Results:**

The expression levels of *BAG-1*, *BAG-2*, *BAG-5* in cancer tissues were significantly downregulated compared with matched pericancerous lung tissues and were protective factors of lung adenocarcinoma (*P* < 0.05, OR < 1); while the expression level of *BAG-4* in cancer tissues were remankably upregulated compared with the matched pericancerous lung tissues and was risk factor of lung adenocarcinoma (*P* < 0.05, OR > 1).

**Conclusion:**

*BAG-1*, *BAG-2*, *BAG-5* might be the potential protective factors while *BAG-4* is possible risk factor of lung adenocarcinoma.

肿瘤的发生发展是一个多因素、多阶段的复杂过程，在基因水平上明确恶性肿瘤的发生发展并寻求治疗手段，是近年来的研究热点。其中，肿瘤细胞凋亡障碍及其机制受到了许多研究者的关注。*BAG*基因（Bcl- 2-associated athanogene）家族是近年发现的抗凋亡基因家族，其分泌蛋白是已经被确认的多功能结合蛋白，可与Bcl-2和热休克蛋白70（heat shock proteins 70, Hsc70/Hsp70）结合，并可以与一系列转录因子作用调节各种生理过程。现有的研究表明，BAG家族成员的表达与多种肿瘤的发病及预后相关。本研究采用基因表达谱芯片技术检测*BAG*家族基因在29例肺腺癌及癌旁组织中的表达，并通过两组GEO数据库中已有的数据进行验证，探讨*BAG*家族基因表达与肺腺癌易感性的关系，为肺癌的发病及预后判断等提供一定的依据。

## 1 材料与方法

### 1.1 材料

#### 1.1.1 临床标本及验证数据

本研究29例患者的肺腺癌组织和距癌组织5 cm外远癌组织配对标本来源于天津医科大学总医院肺外科手术切除标本。其中男性18例，女性11例；年龄最大者75岁，最小者51岁，平均年龄（62.14 ±6.48）岁；组织标本符合以下标准：①患者临床诊断均为Ⅰ期-Ⅳ期（PTNM）原发性肺癌；②术前均未进行化疗和放疗；③手术切除组织立即置入液氮中低温冷冻保存。

验证数据为美国国立生物信息中心（NCBI）GEO数据库（http://www.ncbi.nlm.nih.gov/geo/）下载的分别由LiJen Su和Landi MT等提交的基因表达谱芯片数据GSE7670和GSE10072。GSE7670数据为27例中国台湾省肺腺癌患者，其中男性5例，女性22例；GSE10072数据为33例美国肺腺癌患者，其中男性22例，女性11例；共120张配对的芯片扫描图像文件。

#### 1.1.2 基因芯片

本实验基因表达谱芯片采用美国Affymetrix公司提供的人类基因组HG-U133Plus-2.0芯片，该芯片涵盖了大约54 000个人类基因序列。GEO数据库GSE7670和GSE10072均采用Affymetrix公司HG-U133A芯片，此芯片涵盖大约22 300个人类基因序列。

### 1.2 方法

#### 1.2.1 cRNA的获取及芯片杂交

按照Invitrogen公司操作按明书应用Trizol方法抽提肺腺癌及癌旁组织的总RNA，紫外分析和变性胶电泳检测总RNA质量及浓度，用RNeasy Midi Kit（Qiagen）纯化。按照Affymetrix one-cycle cDNA合成试剂盒实验说明书（2006）进行cDNA合成纯化，以IVT试剂盒进行cRNA合成纯化，之后cRNA片段化，经琼脂糖电泳和紫外分光光度计对片段化的cRNA质控后，进行探针标记及芯片杂交和洗脱，利用Affymetrix公司7G扫描仪进行芯片荧光扫描。以GCOS（gene-chip operation software）软件收集所得数据。

#### 1.2.2 GEO数据库

GSE7670和GSE10072按照Affymetrix表达谱芯片实验说明书（2001）对癌及癌旁组织进行总RNA抽提纯化，cDNA合成纯化，cRNA合成纯化，cRNA片段化质控，探针标记及芯片杂交和洗脱，利用Affymetrix公司6G扫描仪进行芯片荧光扫描。

#### 1.2.3 结果分析

通过Bio-conductor软件对本实验58张和验证数据120张Affymetrix配对的芯片扫描图像文件进行分析及标准化，得到定量及定性信号值，将有效值成组输入统计软件库中进行*Cox*回归拟合单因素条件*Logistic*回归分析。

### 1.3 统计学处理

应用SPSS 16.0软件包进行统计分析，信号值数据以Mean±SD表示，经检验每组信号值均呈正态性分布，采用配对*t*检验法，以*P* < 0.05为差别有统计学意义；并用*Cox*回归拟合单因素条件*Logistic*回归，以*BAG*家族各基因的比值比（OR）及其95%可信区间（CI）表示相对危险度。*P* < 0.05为差别有统计学意义，OR < 1为保护因素，OR > 1为危险因素。

## 2 结果

### 2.1 29例本院肺腺癌患者癌组织及癌旁组织中*BAG*家族基因的表达及其与肺癌易感性的关系（表 1）

**1:** *BAG*家族基因表达与29例肺腺癌的关系 Relationship between the expression of *BAG* family genes and 29 patients of lung adenocarcinoma

Gene	Probe ID	*t* test				*Cox* regression		
		Cancer	Normal	*P*		χ^2^	*P*	OR(95%CI)
*BAG-1*	202387_at	8.648±0.562	8.909±0.349	0.012^*^		8.104	0.004^*^	0.011(0.001-0.248)
	211475_s_at	8.702±0.576	9.013±0.273	0.004^*^		8.017	0.005^*^	0.010(0.000-0.243)
	229720_at	6.746±0.843	7.165±0.556	0.008^*^		5.782	0.016^*^	0.250(0.081-0.774)
*BAG-2*	209406_at	8.225±0.797	8.141±0.465	0.515		6.657	0.010^*^	0.235(0.078-0.706)
	230879_at	4.811±0.489	4.567±0.306	0.004^*^		2.532	0.112	4.230(0.716-24.990)
*BAG-3*	217911_s_at	10.026±0.522	9.849±0.562	0.077		0.015	0.904	1.072(0.347-3.309)
*BAG-4*	222909_s_at	6.498±0.963	5.505±0.565	3.4E-09^*^		6.705	0.010^*^	22.737(2.137-241.942)
	228189_at	9.283±0.670	8.546±0.294	5.9E-09^*^		0.201	0.654	1.303(0.409-4.155)
*BAG-5*	202984_s_at	7.696±0.656	7.520±0.542	0.027^*^		0.013	0.909	0.932(0.279-3.115)
	202985_s_at	9.265±0.404	9.352±0.171	0.163		6.871	0.009^*^	0.010(0.000-0.309)
	230427_s_at	5.868±0.509	5.935±0.385	0.467		6.776	0.009^*^	0.002(0.000-0.215)
^*^P < 0.05.								

*BAG*家族基因中*BAG-1*（探针202387_at、211475_s_at、229720_at）、*BAG-2*（探针209406_at）、*BAG-5*（探针202985_s_at、230427_s_at）基因为肺腺癌易感性的保护因素（*P* < 0.05, OR < 1），且*BAG-1*在正常肺组织中的表达显著高于癌组织（*P* < 0.05），正常肺组织中*BAG-2*、*BAG-5*的表达略高于癌组织；*BAG-4*（探针222909_s_at、228189_at）基因为肺腺癌易感性的危险因素（*P* < 0.05, O R > 1）且在癌组织中的表达显著高于正常肺组织（*P* < 0.05）；*BAG-3*（探针217911_s_at）基因表达与肺腺癌易感性的关系无统计学意义（*P* > 0.05）。对29例肺腺癌患者癌组织*BAG*家族基因表达水平与性别和年龄分别做卡方检验，发现*BAG*家族各基因表达与肺腺癌患者的性别和年龄的关系均无统计学意义（*P* > 0.05）。

### 2.2 GSE7670数据库（表 2）

**2:** *BAG*家族基因表达与27例肺腺癌的关系 Relationship between the expression of *BAG* family genes and 27 patients of lung adenocarcinoma

Gene	Probe ID	*t* test				*Cox* regression		
		Cancer	Normal	*P*		χ^2^	*P*	OR(95%CI)
*BAG-1*	202387_at	7.789±0.537	8.215±0.397	0.006^*^		5.412	0.020^*^	0.207(0.055-0.780)
	211475_s_at	7.571±0.628	8.071±0.323	0.001^*^		6.457	0.011^*^	0.135(0.029.633)
*BAG-2*	209406_at	4.478±0.465	4.731±0.501	0.054		3.186	0.074	0.286(0.072-1.131)
*BAG-3*	217911_s_at	8.092±0.723	8.230±0.667	0.428		0.642	0.423	0.697(0.288-1.686)
*BAG-4*	219624_at	3.633±0.181	3.594±0.106	0.228		1.397	0.237	21.529(0.133-3.493E3)
*BAG5*	202984_s_at	4.391±0.363	4.262±0.189	0.101		2.044	0.153	10.651(0.416-272.740)
	202985_s_at	8.165±0.291	8.322±0.317	0.022^*^		4.208	0.040^*^	0.059(0.004-0.881)
^*^P < 0.05.								

27例中国台湾肺腺癌患者癌组织及癌旁组织表达谱芯片统计分析结果显示：*BAG*家族基因中*BAG-1*（探针202387_at、211475_s_at）、*BAG-5*（探针202984_s_at、202985_s_at）基因为肺腺癌易感性的保护因素（*P* < 0.05, OR < 1）且在正常肺组织中的表达显著高于癌组织（*P* < 0.05），与本研究结果一致；*BAG-2*（探针209406_at）、*BAG-3*（217911_s_at）、*BAG-4*（探针219624_at）基因表达与肺腺癌易感性的关系无统计学意义（*P* > 0.05）。

### 2.3 GSE10072数据库（表 3）

**3:** *BAG*家族基因表达与33例肺腺癌的关系 Relationship between the expression of *BAG* family genes and 33 patients of lung adenocarcinoma

Gene	Probe ID	*t* test				*Cox* regression		
		Cancer	Normal	*P*		χ^2^	*P*	OR(95%CI)
*BAG-1*	202387_at	8.899±0.378	9.222±0.206	7.5E-06^*^		9.305	0.002^*^	0.009(0-0.183)
	211475_s_at	8.593±0.456	9.004±0.325	3.5E-06^*^		9.028	0.003^*^	0.046(0.006-0.344)
*BAG-2*	209406_at	6.960±0.784	6.731±0.630	0.137		2.109	0.146	1.874(0.803-4.372)
*BAG-3*	217911_s_at	9.496±0.517	9.442±0.595	0.674		0.183	0.669	1.233(0.472-3.222)
*BAG-4*	219624_at	5.171±0.548	5.143±0.196	0.771		0.087	0.767	1.215(0.334-4.415)
*BAG5*	202984_s_at	6.239±0.335	6.171±0.460	0.485		0.498	0.480	1.582(0.443-5.654)
	202985_s_at	9.830±0.371	9.881±0.225	0.362		0.838	0.360	0.348(0.036-3.333)
^*^P < 0.05.								

33例美国肺腺癌患者癌组织及癌旁组织表达谱芯片统计分析结果显示：*BAG*家族基因中*BAG-1*（探针202387_at、211475_s_at）基因为肺腺癌易感性的保护因素（*P* < 0.05, OR < 1）且在正常肺组织中的表达显著高于癌组织（*P* < 0.05），与本研究结果一致；*BAG-2*（探针209406_at）、*BAG-3*（217911_s_at）、*BAG-4*（探针219624_at）、*BAG-5*（探针202984_s_at、202985_s_at）基因表达与肺腺癌易感性的关系无统计学意义（*P* > 0.05）。

## 3 讨论

现已发现的*BAG*家族基因成员包括*BAG-1*、*BAG-2*、*BAG-3*、*BAG-4*、*BAG-5*、*BAG-6*，这些多功能结合蛋白也被称为分子伴侣蛋白，通过其保守的BAG结构域与热休克蛋白70（heat shock proteins 70, Hsc70/Hsp70）的ATP酶结构域结合，调节这些伴侣蛋白的功能，参与包括细胞凋亡、肿瘤发生、神经分化、应激反应和细胞周期等各种生理过程。*BAG*家族基因可以正性调节也可以负性调节Hsc70/Hsp70的功能。Hsc70/Hsp70的ATP酶结构域与*BAG*基因结合，它们的底物结合区依然可以结合底物蛋白（如c-Fos、Bcl-2等），*BAG*基因的许多功能如抑制凋亡、对核激素受体的作用以及调控转录等都依赖于Hsc70/Hsp70对其他蛋白的调节作用，这些功能在肿瘤发生发展和预后中起着重要作用。

近年研究中除了BAG-1以外，其他BAG家族成员在肺癌中的表达也少有报道。一般认为BAG-1在肿瘤细胞中的过表达与其临床预后相关。一些研究认为肿瘤细胞浆中BAG-1的过表达降低了肿瘤患者死亡危险性，并可以作为临床良好预后指标。现有的研究^[1, 2]^表明，BAG-1在乳腺癌、肺癌和宫颈癌等多种肿瘤中呈高表达，且BAG-1的高表达与肿瘤患者的临床预后有关，但是过表达的BAG-1在细胞中的分布与肿瘤发生之间的关系还有争议。Tang等^[2]^报道，在140例乳腺癌中，77.7%的乳腺癌BAG-1呈高表达，且BAG-1的高表达与患者的预后不良相关。但在肺癌的研究中，BAG-1的高表达却与患者的良好预后相关^[1]^。我们的研究却表明，在非小细胞肺癌中，BAG-1的表达与患者的预后不良相关，BAG-1低表达组患者生存期明显长于高表达组患者^[3]^。研究结果的差异可能与选用病例的疾病分期及组织学类型不同有关。BAG家族其他成员因为发现较晚，对其功能的研究还不多见。但是，由于它们都具有相同的结构域和相似的结构，都通过BAG结构域与Hsc70/Hsp70等发生作用，故推测它们也参与了肺癌的发生和发展。

本研究通过对29例肺腺癌患者的癌组织及癌旁组织*BAG*家族基因表达的检测发现*BAG-1*、*BAG-2*、*BAG-5*为肺腺癌发生的保护性因素，并在GEO数据库中得到了部分验证。Turner等^[4]^发现122例浸润性乳腺癌组织与正常乳腺组织比较，其中79例BAG-1表达上调，在早期乳腺癌细胞浆中BAG-1的高表达与远处转移和患者生存期呈负相关，提示BAG-1表达的改变可能是早期乳腺癌的一个征兆。其分子机制尚不明确，可能是BAG家族其他成员蛋白与BAG-1竞争结合HSC70/HSP70有关，所以*BAG-1*基因与肿瘤发生之间的关系还有待于进一步研究。

本研究中BAG-2、BAG-5亦为肺腺癌发生的保护因素，Wang等^[5]^在对甲状腺癌中BAG-2的表达研究发现BAG-2表现促凋亡作用，且其促凋亡活性可能为蛋白酶体抑制剂所诱导。BAG-5除了可与Hsc70/Hsp70结合以外，其功能报道甚少。然而，最近的一项研究表明，BAG-5在帕金森氏病中发挥着重要作用。研究人员^[6]^发现BAG-5可抑制E3类酶和Hsc70/Hsp70的活性，促进多巴胺神经细胞变性、凋亡，所以BAG-5可能会在帕金森氏病靶向治疗中起到重要作用。这可能与BAG-2、BAG-5抑制肿瘤发生有一定的关系。另外，本研究结果与GEO数据库结果并不完全一致，有可能与人种、生存环境的差异较大有关。

Liao等^[7]^和Ozawa等^[8]^利用原位杂交和免疫组织化学方法对胰腺癌患者组织标本和胰腺癌细胞株进行检测发现BAG-3和BAG-4均有过表达现象，然而正常胰腺组织中呈低表达，结果显示，BAG-3和BAG-4抑制凋亡作用可能与肿瘤的侵袭生长有关。这与本研究中BAG-4表现为肺腺癌的危险因素的结果相一致。BAG-4的功能与TNF-R1和死亡受体-3（DR-3）的胞浆部分有关。最近的数据^[9]^显示TNF-R1的死亡域N末端含有一个ATP酶结构域，ATP在BAG-4和TNF-R1之间起着调节作用。研究发现在体外Hsc70/Hsp70浓度的增加打断了BAG-4和TNF-R1的连接，这表明Hsc70/Hsp70与TNF-R1竞争结合BAG-4。正常生理状态下，BAG-4与TNF-R1结合，当细胞受到TNF-α刺激时，BAG-4与TNF-R1解离，TNF-R1活化，继而导致凋亡的发生。因此，BAG-4通过与TNF-R1结合发挥抗凋亡蛋白的功能，阻止配体非依赖性寡聚化和受体的活化。在多种癌细胞系中，BAG-4的过表达抑制了TNF-R1诱导的NF-κB的活化和细胞死亡^[8, 10]^。近年也有研究^[11]^发现，细胞浆中BAG-4的高表达与上皮性卵巢癌的侵袭浸润呈负相关。在宫颈癌Hela细胞中BAG-3表现出与Caspases和蛋白酶体相关的抗肿瘤凋亡活性^[12]^。

综上所述，*BAG*家族基因所表达蛋白能够通过其结构域与其他蛋白作用，并通过增加目标蛋白的分子伴侣发挥衔接分子的作用，进而改变这些目标蛋白的功能，其可能参与了包括蛋白降解、细胞增殖、迁移和凋亡等在内的多种细胞生理活动。而很多研究者对*BAG*家族基因在肿瘤细胞中的表达位置及表达量与其在肿瘤发生中的作用还存在不同的观点，*BAG*家族基因的作用还有待进一步研究，探讨其潜在的临床意义，*BAG*家族基因有望作为癌症早期诊断、判断预后、个体化治疗以及靶向治疗的靶分子。
